# Effect of an exercise intervention on global cognition after transient ischemic attack or minor stroke: the MoveIT randomized controlled trial

**DOI:** 10.1186/s12883-022-02805-z

**Published:** 2022-08-04

**Authors:** Inger A. Deijle, Roelofjan Hemmes, H. Myrthe Boss, Edwin C. de Melker, Bob T. J. van den Berg, Gert Kwakkel, Erwin van Wegen, Wendy M. Bosboom, Henry C. Weinstein, Sander M. van Schaik, Renske M. Van den Berg-Vos

**Affiliations:** 1 Department of Neurology, OLVG, Amsterdam, The Netherlands; 2 Department of Physical Therapy, OLVG, Amsterdam, The Netherlands; 3 Department of Neurology, Gelderse Vallei Hospital, Ede, The Netherlands; 4 Department of Cardiology, OLVG, Amsterdam, The Netherlands; 5 Department of Respiratory Medicine, OLVG, Amsterdam, The Netherlands; 6 Department of Rehabilitation Medicine, Amsterdam UMC, location Vrije Universiteit Amsterdam, De Boelelaan 1118, Amsterdam, The Netherlands; 7 Amsterdam Movement Sciences, Rehabilitation and Development, Amsterdam, The Netherlands; 8 Amsterdam Neurosciences, Neurovascular Disorders, Amsterdam, The Netherlands; 9 Department of Physical Therapy and Human Movement Sciences, Northwestern University, Chicago, IL USA; 10 Department of Neurorehabilitation, Amsterdam Rehabilitation Research Centre Reade, Amsterdam, The Netherlands; 11 Board of directors, Rivierenland Hospital, Tiel, The Netherlands; 12 Department of Neurology, Amsterdam UMC, location AMC, Amsterdam, The Netherlands

**Keywords:** Ischemic stroke, Transient ischemic attack, Cognition, Physical fitness training

## Abstract

**Background:**

Patients with a transient ischemic attack (TIA) or ischemic stroke are at increased risk of developing cognitive impairment in the subacute phase. At present, the effects of exercise on cognitive functioning following a TIA or stroke are not fully known. The purpose of this trial was to investigate the effect of exercise on global cognition.

**Methods:**

The MoveIT trial is a single-centre, observer-blinded, randomized controlled trial involving a 1-year exercise intervention consisting of a 12-week group exercise program, combined with three counselling visits to the physiotherapists over a 9-month period. The control group received standard care. The primary outcome was global cognitive functioning, assessed at one year, using the Montreal Cognitive Assessment (MoCA). Secondary outcomes included cardiorespiratory fitness, the cardiovascular profile, and attainment of secondary prevention targets, anxiety, depression and fatigue at one and two years.

**Results:**

The experimental group consisted of 60 patients, while the control group consisted of 59 patients. The mean age was 64.3 years and 41% were female. No between-group differences were found on global cognitive functioning (MD, 0.7 out of 30, 95% CI, − 0.2 to 1.6) or on secondary outcome measures at 12 months. The only significant between-group difference was found for fatigue, in favour of the experimental group at 12 months (MD, 0.6 out of 63, 95% CI, 0.1 to 1.1).

**Conclusions:**

No benefit of this exercise intervention was found regarding global cognition. Future studies need to focus on optimizing rehabilitation strategies for this vulnerable group of patients.

**Trial registration:**

http://www.trialregister.nl. Unique identifier: NL3721.

Date first registration: 06-03-2013.

## Introduction

Patients with a transient ischemic attack (TIA) or ischemic stroke are at increased risk of developing cognitive impairment [1, 2]. A systematic review has shown that post-TIA cognitive impairment occurs frequently, with a prevalence of mild cognitive impairment in 29–68% and severe cognitive impairment in 8–22% of patients [3]. Up to 76% of patients with stroke had mild cognitive impairment at 3 months after an acute stroke, although up to 50% of these patients showed cognitive improvement, and 30% show delayed deterioration between 3 and 15 months post stroke [1, 4].

Given the lack of successful pharmaceutical treatments for cognitive decline, alternative treatment strategies to prevent cognitive decline must be investigated. Physical activity and exercise (both aerobic and strength training) are widely accessible, low-cost treatments, which has been associated with improved cognitive functioning in populations at increased risk for cognitive decline, including neurologically healthy older adults and those with dementia [5–7]. We define physical activity as “any bodily movement produced by skeletal muscles that results in energy expenditure,” whereas exercise is “a subset of physical activity that is planned, structured, and repetitive and has as a final or an intermediate objective the improvement or maintenance of physical fitness.” [8] Physical activity and exercise are recommended to stroke survivors to reduce disability, and have beneficial effects on cardiorespiratory health, functional capacity, activities of daily living and quality of life [9]. Unfortunately, the impact of physical activity and exercise on cognitive functioning in patients with TIA or stroke is still unclear, and investigation has been urgently recommended [9, 10].

Two recent meta-analyses showed inconclusive results, but both suggested a potential benefit of an exercise intervention on attention and processing speed in patients with stroke [11, 12]. A recent report using an exercise intervention noted improvements in overall cognition and in the subdomains of attention, concentration, visuospatial and executive functioning [13]. Another observational study in patients with varying degrees of cerebral small vessel disease found a reduction in the risk of cognitive impairment and dementia in patients who were more physically active [14].

Previously, we demonstrated in a pilot trial the safety and feasibility of an exercise intervention in the subacute phase after a TIA or minor ischemic stroke [15]. Since prevention of cognitive decline after stroke is recognized as an important goal, and evidence-based strategies are still lacking, the aim of the present trial was to investigate the effect of a 1-year exercise intervention on cognition in patients after a TIA or minor ischemic stroke.

## Methods

### Study design

The MoveIT trial was a single-centre, observer-blinded, randomized controlled trial. Patients were randomized in permuted blocks, using sealed opaque envelopes to conceal treatment allocation. The envelopes were prepared and secured by an independent physician. The outcome measures were recorded on standardized forms by trained observers blinded to the treatment allocation. The control group received usual care for patients after TIA or minor ischemic stroke, consisting of two or three visits to the outpatient clinic. The follow-up period was 2 years after the initial TIA or stroke, and the last patient was assessed in October 2016. All methods in the MoveIT trial were carried out in accordance to the Declaration of Helsinki. Study procedures were approved by local university and hospital research ethics committees, location VU Medical Centre, NL38008.029.11. Written informed consent was obtained from all patients. The study protocol was published in December 2014 in BMJ Open [16]. The trial has been registered in the Netherlands Trial Register with the registration number NL3721 and was first registered at 06-03-2013. Our reporting follows the CONSORT statement for reporting parallel-group randomized trials (http://www.consort-statement.org/consort-statement/flowdiagram).

### Subjects

Patients were invited to take part in the study by clinicians at the stroke unit, emergency department and outpatient clinic of our secondary care teaching hospital. They were screened for eligibility and consented between April 2012 and June 2014. Patients were eligible if they 1) were at least 18 years old, 2) presented with a TIA or minor ischemic stroke as defined by a National Institutes of Health Stroke Scale (NIHSS) score ≤ 3, 3) had had the onset of signs and symptoms less than 1 month ago, 4) were able to walk independently, 5) had been discharged from hospital without need for further rehabilitation, [17] 6) had a Mini-Mental State Examination (MMSE) score ≥ 24, 7) had no aphasia and were able to speak Dutch, 8) had no cardiopulmonary contraindications for physical exercise and exercise testing as outlined by the American College of Sports Medicine, [18] and 9) had no chronic disease with an expected survival of less than 2 years.

### Cardiopulmonary examination

Cardiopulmonary examination was conducted to ensure the exercise intervention was safe in this group of patients. Before randomization, all potential participants were screened for cardiac contraindications using a checklist that included history of cardiac disease, symptoms of current cardiac and pulmonary disease and the results of an electrocardiogram (ECG). Potential participants with a positive checklist were examined by a cardiologist and/or pulmonologist; the randomization was done after their approval. At baseline, all maximum exercise capacity tests (see below) were checked by the cardiologist and pulmonologist, and in case of abnormalities, additional examinations were done. Patients in the experimental group whose tests showed abnormalities started the exercise intervention after completing the additional examination, upon approval by the cardiologist and/or pulmonologist.

### Outcomes

The primary outcome measure was global cognitive functioning, assessed with the Montreal Cognitive Assessment (MoCA) [19] at 1 year post stroke, while the MoCA score at 2 years was a secondary outcome measure. In addition, a standardised neuropsychological examination was conducted at baseline, 1 and 2 year post stroke, which consisted of attention, verbal and visual memory, and executive functioning tests. Cardiorespiratory fitness was measured as maximal exercise capacity or maximal oxygen consumption (V̇O_2max_), measured by performing a ramp exercise test on a cycle ergometer (Jaeger) under continuous blood pressure measurement, ECG, and breath-by-breath gas analysis (Oxycon Pro). The amount of self-reported physical activity was measured using the Physical Activity Scale for the Elderly (PASE) questionnaire [20, 21]. In accordance with international literature concerning the attainment of primary goals in secondary stroke prevention, we used a composite score for optimal therapy, defined as the combination of prescribed antithrombotic therapy (antiplatelet agents or oral anticoagulants) and meeting targets for both blood pressure (< 140/90 mmHg) and low density lipoprotein (LDL) cholesterol (< 100 mg/dl) [22]. Self-reported smoking, alcohol consumption, Body Mass Index (BMI) and waist circumference were also assessed. Mental health was assessed using the Hospital Anxiety and Depression Scale (HADS) [23]. The severity of fatigue was measured using the Fatigue Severity Scale (FSS), a 9-item scale of which the average score was used [24]. All secondary outcomes wre measured at baseline and at 12 and 24 months. 

### Intervention

All patients in this trial received standard post-stroke care according to the Dutch multidisciplinary guideline, [25] consisting of a total of two visits to the outpatient clinic during the first 3 months after the TIA or minor stroke. The patients in the experimental group started the exercise intervention with an intake session in which their current exercise behaviour and motivation were assessed and exercise goals were established. The exercise intervention started with a 12-week exercise program which was performed in groups of 10 patients. The patients received two one-hour sessions of exercise training per week, supervised by two specialized physiotherapists. The exercise program consisted of both aerobic and strength training. The aerobic exercise was performed using a cycle ergometer, treadmill, or rowing machine. The Karvonen formula was used to calculate the target heart rate (THR), as follows: resting heart rate + (% desired intensity x [maximum heart rate derived from the maximum exercise test – resting heart rate]). Aerobic exercise started at 40%, and was then gradually increased to 80% THR [18]. During each aerobic exercise training session, heart rate was measured twice to ensure that the patients exercised at the target level. We also used a rating of perceived exertion of 11 to 16 (“light” to “hard”) on the Borg 6–20 Scale [26]. The strength training part of the program, performed on weight machines, started with 3 sets of 10–12 repetitions at 30% of the repetition maximum, and patients gradually progressed to 60–70%. Patients were instructed and guided to perform home-based exercise sessions three times a week, with the aim of achieving independence in exercising and to develop and maintain an active lifestyle that meets recommended physical activity and exercise guidelines. In order to gain insight in the amount and frequency of physical training, patients used an exercise diary, which recorded how much they exercised and what the facilitators and barriers were for an active lifestyle. In this diary the patients kept track of how much they exercised per day and what were motivators and barriers to exercise. After the completion of the 12-weeks group exercise program, follow-up consisted of three visits to the physiotherapist over a 9-month period. During these follow-up visits, the patients received counselling by the physiotherapists, based on motivational interviewing. Patients were motivated to maintain an active lifestyle and to continue exercising, the exercise diary was used as an evaluation method for the patient. The description of our intervention followed an internationally developed template on exercise reporting [27].

### Power calculation and sample size

Global cognition, assessed with the MoCA test, was selected as the outcome variable to calculate sample size [19, 28]. Using estimates obtained from the literature [29, 30] and our previously performed pilot study, [15] a sample size of 52 patients in each group was needed to reach a power of 80% in detecting a difference in mean MoCA score of 1.5 points, assuming a standard deviation of 2.7 and using a two-sample t test with a 0.05 two-sided significance level. We increased the sample size to 60 patients in each group to anticipate potential dropouts.

### Statistics

Baseline demographic and clinical characteristics of the patients in the experimental and control groups were compared using independent two-sample t-tests (for continuous data) and χ2 analysis (for nominal data). Visual inspection of the distribution was used to assess normality. Primary analyses were unadjusted, following the intention-to-treat principle. The between-group differences in change scores from baseline to 12 months and from baseline to 24 months were investigated using independent t-tests for continuous outcomes. Mann-Whitney non-parametric statistics were used in those cases where assumptions of normality were violated. Mixed-effects regression models were used with a random intercept for longitudinal repeated measures. Each hypothesis was tested with a two-tailed analysis with 0.05 as the level of significance. All analyses were carried out in SPSS, version 22.

The results of the study were shared with all participants with a written summary containing the main outcomes of the study. Patients were not involved in the development, design, recruitment or conduct of the MoveIT study. The burden of the intervention was not assessed by patients themselves.

## Results

**Fig. 1:**
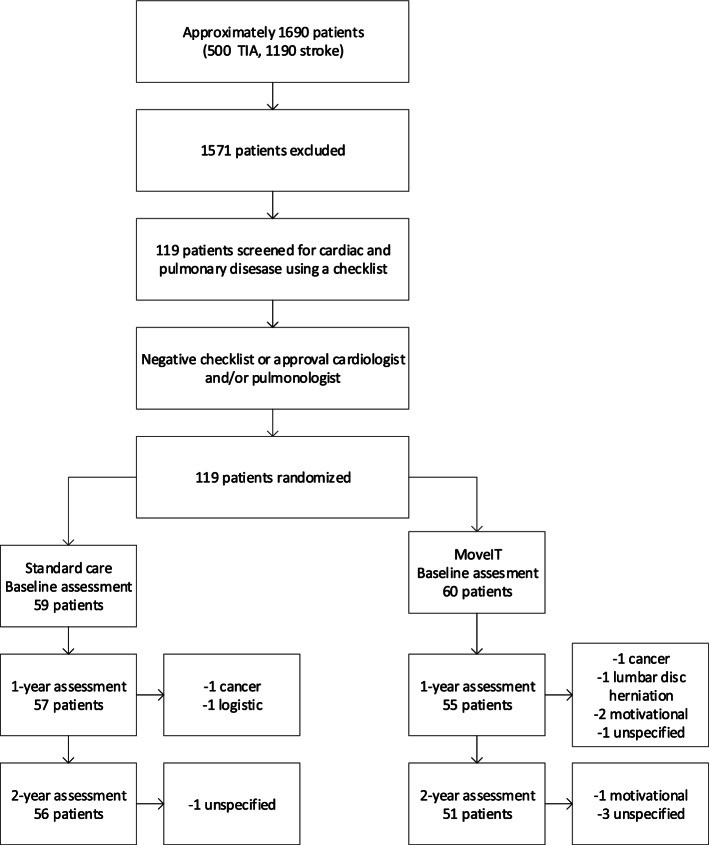
Consort flow chart for patient recruitment

An estimated number of 1690 patients with TIA or stroke (500 TIA and 1190 stroke patients, based on the prospective stroke register) were screened from April 2012 to June 2014 (Fig. 1).

Of the total of 119 patients enrolled in the study, 60 were allocated to the treatment group and 59 to the control group. The main reason for not participating was the intensive nature of the intervention and the measures. In the experimental group, 49 patients (81.7%) completed the 2-year assessment, compared with 51 patients (86.4%) in the control group. There was no significant difference between the experimental and control groups as regards the attrition rate (*P* = 0.47). Baseline characteristics of the study population are shown in Table 1, which shows that there were no significant differences at baseline between the experimental and control groups.

**Table 1:** Baseline data

	Experimental group (***n*** = 60)	Control group (***n*** = 59)
Demographic characteristics		
Male (%)	34 (57)	36 (61)
Age (y) ± SD, range	64.7 ± 8.9, 44–86	63.9 ± 10.6, 44–85
Stroke type		
TIA (%)	34 (57)	27 (56)
Ischemic stroke (%)	26 (43)	32 (54)
Stroke localization		
Left (%)	30 (50)	31 (53)
Right (%)	18 (30)	16 (27)
Vertebrobasilar (%)	12 (20)	11 (19)
Vascular risk factors		
Waist circumference in cm ± SD	96.7 ± 11.2	96.4 ± 10.9
Systolic blood pressure (mmHg) ± SD	124.5 ± 15.0	123.2 ± 15.2
Diastolic blood pressure (mmHg) ± SD	74.9 ± 9.6	74.3 ± 9.6
Hypertension^a^ (%)	48 (80)	44 (73)
Anti-hypertensive treatment (%)	41 (68)	42 (71)
LDL-cholesterol (mmol/l) ± SD	2.3 ± 0.7	2.3 ± 0.7
Hypercholesterolemia (%)	47 (78)	37 (62)
NIHSS, median (IQR)	0 (1)	0 (1)
Active smoking (%)	7 (12)	14 (24)
History of smoking (%)	26 (43)	34 (58)
Alcohol overuse (%)	4 (7)	6 (10)
History of stroke (%)	15 (25)	13 (27)
Atrial fibrillation (%)	0 (0)	2 (3)
History of myocardial infarction or other cardiac ischemia (%)	9 (15)	11 (19)
History of other cardiac illness (%)	49 (82)	50 (85)
History of other cardiovascular disease (%)	42 (70)	40 (68)
Peripheral Artery Disease (%)	1 (2)	2 (3)
Family history of stroke (%)	25 (42)	25 (42)
Diabetes (%)	5 (8)	9 (15)
Other outcomes		
MoCA (0–30) ± SD	24.9 ± 3.2	25.5 ± 2.9
VO_2max_ (ml/kg/min) ± SD	23 ± 6.5	22 ± 6.4
PASE (0–361) ± SD	133.8 ± 64.7	117.5 ± 68.9
HADS (0–42) ± SD	8.3 ± 5.7	10.3 ± 6.6
FSS 1-7 ± SD	3.7 ± 1.5	4.0 ± 1.6
History of COPD or asthma (%)	3 (5)	3 (5)
OSAS (%)	2 (3)	3 (5)
Thrombolysis^b^ (%)	4 (7)	6 (10)
Time from TIA/stroke to randomization, in days (median)	30	21

Values are given as means with standard deviation (SD) with range or as number (percentage), unless otherwise stated.

^a^Based on medical history, use of antihypertensive treatment, or blood pressure > 140/90 mmHg

^b^Having received thrombolysis treatment for the ischemic event at presentation

The median intervals between the TIA or minor stroke and the moment of randomization to the experimental or control group were 30 and 21 days, respectively. The median time from the randomization to the start of the intervention was 63 days, due to additional examination(s) after the baseline maximal exercise capacity test by the cardiologist/pulmonologist. Among the 60 patients in the experimental group, 22 received further examinations, after which they were allowed to start the intervention. No serious adverse events occurred.

### Primary outcome

No between-group differences were found on global cognitive functioning (MD, − 0.7 out of 30, 95% CI, − 1.6 to 0.2) as measured with the MoCA at 12 months (Table 2). The results of the standardised neuropsychological examination on the cognitive domains attention, verbal and visual memory and executive functioning will be published separately.

**Table 2:** Mean (SD) of Groups, and Mean (95% CI) Difference between Groups for All Outcome

	Groups						Difference within groups				Difference between groups	
	Baseline		12 months		24 months		Month 12 Minus baseline		Month 24 Minus baseline		12 months vs baseline	24 months vs baseline
	Exp (***n*** = 60)	Con (***n*** = 59)	Exp (***n*** = 60)	Con (***n*** = 59)	Exp (***n*** = 60)	Con (***n*** = 59)	Exp (***n*** = 60)	Con (***n*** = 59)	Exp (***n*** = 60)	Con (***n*** = 59)	Exp Minus Con	Exp Minus Con
MoCA 0-30	24.9 (3.2)	25.5 (2.9)	25.9 (2.8)	25.9 (3.0)	25.7 (2.8)	26.0 (3.1)	−1.1 (2.4)	−0.4 (2.3)	−0.6 (2.2)	− 0.4 (2.4)	0.7 (− 0.2 to 1.6)	0.2 (− 0.7 to 1.1)
V̇O_2max_ (ml/kg/min)	23.0 (6.6)	22.0 (6.4)	23.1 (6.8)	21.6 (6.0)	22.2 (6.2)	22.4 (6.0)	0.1 (3.0)	−1.0 (3.0)	−0.5 (3.7)	−0.5 (2.9)	−1.1 (−2.3 to 0.1)	0.0 (− 1.4 to 1.5)
PASE 0-361	133.8 (64.7)	117.5 (68.9)	160.6 (81.5)	131.1 (81.6)	154.4 (90.4)	126.6 (83.3)	24.3 (72.8)	22.3 (75.7)	17.6 (80.9)	13.6 (85.6)	−1.7 (−30.7 to 27.3)	−4.0 (−38.3 to 30.3)
Systolic blood pressure (mmHg)	124.5 (15.0)	123.2 (15.2)	124.2 (17.3)	122.0 (13.2)	125.9 (19.6)	124.5 (14.4)	−0.2 (13.6)	−0.9 (12.8)	2.0 (15.2)	1.4 (12.2)	−0.7 (−5.6 to 4.2)	− 0.5 (−6.0 to 5.0)
Diastolic blood pressure (mmHg)	74.9 (9.6)	74.3 (9.7)	75.3 (9.7)	74.3 (8.0)	75.7 (9.6)	74.5 (9.1)	0.5 (6.5)	−0.4 (5.7)	0.8 (7.7)	−0.0 (7.8)	−0.9 (−3.2 to 1.4)	−0.8 (− 3.9 to 2.2)
Total cholesterol (mmol/l)	4.4 (0.8)	4.4 (0.9)	4.5 (1.0)	4.4 (1.0)	4.5 (0.9)	4.4 (0.9)	0.1 (0.8)	0.0 (0.9)	0.1 (0.8)	0.1 (0.7)	−0.1 (−0.4 to 0.2)	−0.1 (− 0.4 to 0.2)
LDL-cholesterol (mmol/l)	2.3 (0.7)	2.3 (0.7)	2.4 (0.8)	2.5 (0.8)	2.4 (0.8)	2.5 (0.8)	0.1 (0.6)	0.1 (0.8)	0.1 (0.6)	0.1 (0.8)	0.0 (−0.3 to 0.2)	0.0 (−0.2 to 0.3)
HADS total (0–42)	8.3 (5.7)	10.3 (6.7)	8.0 (6.2)	9.7 (6.0)	7.4 (5.8)	9.6 (6.4)	−0.1 (4.1)	−0.7 (4.7)	− 0.2 (4.1)	− 0.9 (5.9)	− 0.6 (−2.3 to 1.0)	−0.8 (−2.8 to 1.3)
HADS anxiety (0–21)	4.4 (3.2)	5.6 (3.7)	4.1 (3.2)	4.9 (3.7)	4.0 (3.2)	5.1 (3.4)	−0.2 (1.8)	−0.7 (2.9)	− 0.1 (2.3)	−0.7 (3.6)	− 0.5 (−1.4 to 0.4)	−0.6 (− 1.8 to 0.6)
HADS depression(0–21)	3.9 (3.1)	4.7 (3.6)	3.8 (3.7)	4.8 (3.5)	3.4 (3.2)	4.5 (3.7)	0.1 (2.8)	−0.0 (2.5)	−0.1 (2.8)	− 0.3 (2.8)	−0.2 (−1.2 to 0.8)	−0.2 (− 1.3 to 0.9)
FSS (1-7)	3.7 (1.5)	4.0 (1.6)	3.4 ± 1.3	4.3 ± 1.5	3.4 ± 1.6	4.0 (1.6)	−0.3 (1.2)	0.3 (1.4)	−0.3 (1.1)	0.2 (1.6)	0.6 (0.1 to 1.1)	0.5 (0.0 to 1.0)

*Exp* experimental, *Con* control group

### Secondary outcomes

There were no significant differences between the experimental and control groups in global cognitive functioning as measured with the MoCA at 24 months (Table 2). At baseline, patients in the experimental group had a mean maximal exercise capacity of 23.0 ml/kg/min, compared to 22.0 ml/kg/min in the control group, which is in the 5th percentile of age- and sex-related normative values [18]. The level of fitness was very poor (below the 20th percentile of age- and sex-related normative values) for 86% of the female patients and 61% of the male patients [18]. There were no significant differences between the experimental and control groups in cardiorespiratory fitness at 12 and 24 months (Table 2). We found no between-group difference in the level of self-reported physical activity as measured by the PASE.

No significant difference in the composite score for attainment of optimal secondary prevention targets was found between the study groups. At 12 months, 73% of the patients in the experimental group and 78% in the control group had attained the endpoint of optimal medical therapy, while at 24 months, 60% of the patients in both the experimental and control groups had attained this endpoint. Adherence to antithrombotic medication was 100% in both groups at both 12 and 24 months. We found no between-group differences in systolic blood pressure, diastolic blood pressure, total cholesterol, LDL-cholesterol, weight, waist circumference, or active smoking after 12 and 24 months. The mixed-effects regression model did not found another outcome than the primary analysis (Table 3).

**Table 3:** Between-group linear mixed model results: the experimental group compared with control group

	Linear mixed model	
Variable	Between-group difference in change	***P*** value
MoCA	−0.02	0.67
V̇O_2max_(ml/kg/min)	1.81*	0.05*
PASE	21.1	0.08
Systolic blood pressure (mmHg)	1.6	0.53
Diastolic blood pressure (mmHg)	1.2	0.47
Total cholesterol (mmol/l)	0.01	0.97
LDL-cholesterol (mmol/l)	−0.1	0.64
HADS total	−1.7	0.11
HADS anxiety	−0.9	0.11
HADS depression	−0.8	0.20
FSS	−0.6	0.01

*Corrected for age and sex

There were no significant between-group differences in the total HADS, HADS Anxiety and HADS Depression scores after 12 and 24 months. The experimental and control groups both had a mean HADS anxiety score at baseline of 4.4 ± 3.2 respectively 5.6 ± 3.7 and mean HADS depression score at baseline of 3.9 ± 3.1 respectively 4.7 ± 3.6. A score of > 8 indicating a possible anxiety disorder or depression applies per subscale [23]. The only significant between-group difference was found for fatigue, in favour of the experimental group at 12 months (Mean Difference (MD), − 0.6 out of 63, 95% CI, 0.1 to 1.1). This mean difference had become non-significant at 24 months (MD, 0.5 out of 63, 95% CI, 0.0 to 1.0).

### Recurrent vascular disease

We found no significant between-group differences in the number of deaths, recurrent TIAs or strokes at 12 and 24 months post stroke. At 24 months, new vascular events had occurred in 7 patients in the experimental group and 15 in the control group (*p* = 0.10). Two patients in the experimental group and 6 in the control group had a recurrent TIA or ischemic stroke (*p* = 0.27).

### Intervention participation

Of the 60 patients in the experimental group, 43 completed the exercise intervention. One patient withdrew from the trial before the intervention started, 8 patients completed the group exercise program of 12 weeks but did not attend the 3 counselling visits thereafter and 8 patients neither completed the group exercise program nor the counselling. Reasons for not completing the exercise intervention were: severe osteoarthritis (*n* = 2), financial problems (*n* = 1), lack of time (*n* = 2), no reason (*n* = 12).

## Discussion

The MoveIT trial found no additional benefit of a 1-year exercise intervention compared to usual care, regarding our primary outcome of global cognitive functioning at 1 year, as measured with the MoCA. In addition, we found no significant between-group differences in secondary outcomes like cardiorespiratory fitness, the attainment of secondary prevention targets and self-reported measures of anxiety and depression. The only significant between-group difference was found for fatigue, which was less in the experimental group than in the control group at 12 months. The neutral outcome of our trial suggests that an exercise intervention comprising of a 12-week group exercise program and a 9-months follow-up, both under the guidance of specialized physiotherapists, has no favourable effect on global cognitive functioning.

An explanation for the lack of effect on global cognition functioning might be that patients were relatively young, the proportion of patients with TIA was relatively high (50%), and their cognition was relatively good, with baseline MoCA scores approaching normal values. The cognitive decline in our included patients may have been too small to produce a measurable effect. Furthermore, we may have missed the limited time window of enhanced neuroplasticity characterized by an upregulation of growth-promoting factors such as BDNF [31]. This window, which is present within the first days or weeks after a stroke, may have been missed as we started our intervention later due to the thorough prior cardiopulmonary examinations.

There were no significant between-group differences in cardiorespiratory fitness at 12 and 24 months, indicating there was no training effect. In recent studies both moderate and high intensity training increased V̇O_2max_, but the V̇O_2max_ was measured immediately after the training period lasting 6 months and 3 months, respectively [32–34]. Long-term effects were not measured in these three studies. Since we were interested in long-lasting effects of an exercise intervention on cognition, we chose to measure the long-term effects and did not perform measurements of cognitive functioning or physical fitness immediately after participants had completed the exercise group training. Because during each exercise training, heart rate was measured twice to ensure that the patients exercised at the target level, we think the intensity of the training was sufficient to allow improvement of physical fitness.

This may mean that a possible intervention effect on cognition and cardiorespiratory fitness in our trial may have disappeared. The explanation for the lack of effect may possibly be found in the fact that the intervention did not succeed in maintaining an active lifestyle after the 12 week training program. Future trials should consider an earlier start of the intervention, with a less comprehensive cardiopulmonary examination, and a higher dosage in terms of frequency and duration of the exercise intervention, to establish whether an exercise intervention can indeed improve cognitive functioning.

It must be emphasized that the baseline cardiorespiratory fitness in both the experimental and control group was very poor, which confirms the results of our previously conducted pilot study [15]. We do not know precisely why the cardiorespiratory fitness of our patients was so poor. In the pilot study we have hypothesized that the poor fitness was due to premorbid cardiovascular and pulmonary disease and vascular risk factors, but not to stroke-related factors like weakness or limb coordination problems [35]. We did not exclude patients with possible anxiety and depression disorders, which are likely to impact on the ability to increase their physical activity level in the long term [36]. Although, in our trial the mean HADS anxiety and HADS depression in both groups were not above the already described cut off points of 8, 20% had a HADS score ≥ 8 on the HADS anxiety and 17% of the patients had a score ≥ 8 on the HADS depression. The percentage in our study for anxiety was slightly lower than in another study, which reported a slightly higher prevalence of anxiety in 24% of minor stroke patients, [37] another study found a much lower incidence of 6% [38]. For depression the percentage of 17% in our study is higher than other studies have reported, namely 12% for patients with TIA [39, 40] and 6% for patients with minor stroke [37]. Because anxiety and depression affects the improvement of physical activity and has an incidence of 20 and 17% respectively in patients with TIA and minor stroke, we recommend that future research should focus on the occurrence and treatment of depression and anxiety during an exercise intervention. Possibly, anxiety and depression must be treated first, or simultaneously with the exercise intervention, to make sure the intervention has an effect on exercising and physical activity in patients with depression and anxiety.

The first limitation is the delayed start of the intervention at a median of 3 months after the TIA or minor stroke, for safety reasons. Second, we did not measure the short-term effects of the 12-week exercise program, which leaves it unclear if the lack of effect on cardiorespiratory fitness was due to the intensity of the intervention or to the moment of measuring. Third, the drop-out rate of patients shortly after inclusion was relatively high, which may have led to an underpowered study. The estimated MoCA score of 1.5 used in the power calculation matches the recently reported minimal clinically important difference of between 1.2 and 2.2 found in a stroke population [41]. Unfortunately, our power analyses may not have taken enough account of the high dropout rate. Apparently, this intervention and the assessments represented a burden for patients, a notion which is important for future research on this topic.

## Conclusions

The current study found no benefit of physical fitness training on global cognitive functioning in the subacute phase after a TIA or minor stroke. We found that baseline cardiorespiratory fitness in our patients with TIA or minor stroke was very poor, a finding which may have implications for future studies in which we hope to define optimal rehabilitation strategies for this vulnerable group. Given the profound impact of post-stroke fatigue on the quality of life, [42, 43] and the positive effect of our intervention on fatigue, further studies are needed to elucidate the mechanisms of this effect.

## Data Availability

The datasets used and/or analysed during the current study are available from the corresponding author on reasonable request.

## Abbreviations

TIA: Transient ischemic attack
NIHSS: National Institute of Health Stroke Scale
MMSE: Mini-Mental State Examination
ECG: Electrocardiogram
V̇O_2max_: Maximal oxygen consumption
MoCA: Montreal Cognitive Assessment
PASE: Physical Activity Scale for the Elderly
LDL: Low density lipoprotein
BMI: Body mass index
HADS: Hospital Anxiety and Depression Scale
FSS: Fatigue Severity Scale
THR: Target exercise heart rate
IQR: Interquartile Range
COPD: Chronic Obstructive Pulmonary Disease
OSAS: Obstructive Sleep Apnoea Syndrome
